# Disease-Specific Prediction of Missense Variant Pathogenicity with DNA Language Models and Graph Neural Networks

**DOI:** 10.3390/bioengineering12101098

**Published:** 2025-10-13

**Authors:** Mohamed Ghadie, Sameer Sardaar, Yannis Trakadis

**Affiliations:** 1 Research Institute of the McGill University Health Centre (RI-MUHC), Montreal, QC H4A 3J1, Canada; 2 Department of Human Genetics, McGill University, Montreal, QC H3A 0G4, Canada; 3 Department of Medical Genetics, McGill University Health Center, Montreal, QC H4A 3J1, Canada

**Keywords:** machine learning (ML), neural network classifier, variants of uncertain significance (VUS), missense variants, disease-specific variant interpretation, genetic variant pathogenicity prediction, ClinVar, graph convolutional neural network (GCN), genomic embeddings

## Abstract

Accurate prediction of the impact of genetic variants on human health is of paramount importance to clinical genetics and precision medicine. Recent machine learning (ML) studies have tried to predict variant pathogenicity with different levels of success. However, most missense variants identified on a clinical basis are still classified as variants of uncertain significance (VUS). Our approach allows for the interpretation of a variant for a specific disease and, thus, for the integration of disease-specific domain knowledge. We utilize a comprehensive knowledge graph, with 11 types of interconnected biomedical entities at diverse biomolecular and clinical levels, to classify missense variants from ClinVar. We use BioBERT to generate embeddings of biomedical features for each node in the graph, as well as DNA language models to embed variant features directly from genomic sequence. Next, we train a two-stage architecture consisting of a graph convolutional neural network to encode biological relationships. A neural network is then used as the classifier to predict disease-specific pathogenicity of variants, essentially predicting edges between variant and disease nodes. We compare performance across different versions of our model, obtaining prediction-balanced accuracies as high as 85.6% (sensitivity: 90.5%; NPV: 89.8%) and discuss how our work can inform future studies in this area.

## 1. Background

Predicting the impact of genetic variants in human genetics has gained wide attention in the past two decades [1]. With advancements in machine learning (ML) techniques, recent efforts have focused on developing ML models for variant interpretation. Saloom et al. made use of sophisticated deep learning classification techniques to predict the type and influence of genetic variants on an individual’s risk to develop disease [2]. Yang et al. used a gradient boosting decision tree to predict pathogenicity of structural variants after annotating them with genomic, proteomic, and epigenomic features [3]. Liu et al. applied a gradient boosting model on nonsynonymous variants annotated with 60 features from 6 categories including epigenomics, functional effect, splicing effect, population-based features, biochemical properties, and conservation [4]. Similarly, Molotkov et al. proposed an ensemble model for predicting pathogenicity of nonsynonymous variants [5]. Furthermore, Ge et al. used epigenomic functional and evolutionary information extracted from genome and protein sequences to predict pathogenicity of missense variants [6].

Other studies applied different graphical approaches to integrate genetic variant information with other biological data. Zhang et al. used a graph attention neural network to predict pathogenicity of missense variants, with graph nodes capturing predictive features of amino acids and edges weighted by strength of feature co-evolution [7]. Cheng et al. predicted pathogenicity of missense variants using a graph neural network operating on a network of genes and variants [8]. Wang et al. mapped atomic connections in protein 3D structures to residue-level network representations and used structural, topological, biophysical and sequence properties of mutation sites as node attributes to predict variant pathogenicity using a graph neural network [9]. These graph-based approaches mainly rely on biomolecular, structural and evolutionary information. Other graph-based studies such as Kamada et al. made use of higher-level aspects such as pathways to predict the pathogenicity of missense variants [10]. Hou applied a graph neural network on a graph of protein sequences passed through an Evolutionary Scale Modeling (ESM) model to predict pathogenicity of nonsynonymous mutations [11].

These studies rely on an ensemble of techniques that require processing and integrating biological data from many different sources. For example, the performance of the ML model by Cheng et al. [8] relies heavily on pathogenicity scores obtained from PrimateAI [12]. PrimateAI uses protein 3D structures, multiple sequence alignments and variant frequencies across primate species to infer variant pathogenicity. Moreover, the aforementioned studies have focused on predicting pathogenicity of variants in a disease agnostic (non-disease-specific) context. However, interpreting a variant in the context of a specific disease can be more clinically useful, as it allows for integration of disease-specific domain knowledge. Zhan and Zhang provided a neural network framework that can be fine-tuned on genomic foundation models for more accurate prediction of variant pathogenicity in a disease-specific context [13]. They evaluated their model in the context of cardiovascular disease and post-transcriptional regulation of RNA splicing.

Despite all efforts to date, most missense variants identified on a clinical basis are classified as variants of uncertain significance (VUS) [14]. Our study aims to advance the field of variant pathogenicity prediction for missense variants, by combining different key strategies. First, we follow a graph-based approach that not only includes information at the molecular level but also information on higher-level aspects of human biology such as phenotype, disease, drug and clinical information. Second, we integrate large-scale structural, functional, and evolutionary data by taking advantage of DNA language models. In brief, similar to ESM pretrained models for proteins, DNA language models can capture variant features directly from its genomic sequence: e.g., DNABERT [15], HyenaDNA [16] and Nucleotide Transformer [17]. Third, we follow the more clinically useful disease-specific approach for predicting variant pathogenicity by utilizing our knowledge graph to predict whether a variant is pathogenic or benign, in relation to a specific disease.

## 2. Methods

### 2.1. Graph-Based Prediction of Disease-Specific Variant Pathogenicity

We obtained a heterogeneous biomedical knowledge graph from Chandak et al., 2023 [18] consisting of 129,375 nodes representing 10 different aspects of human biology, clinical, and environmental information. The 10 node types are protein, disease, drug, phenotype, pathway, molecular function, biological process, cellular component, drug, exposure, and anatomy. These nodes are connected through 8,100,498 edges belonging to 30 different types. To integrate variants associated with genes into the graph, we first split each protein node into two nodes, a gene node and a protein node, with each protein connected to its corresponding gene through a new type of edge. We then also connected each variant to its associated gene through a new type of edge. In addition, each pathogenic variant was connected to the disease it is known to be associated with in the ClinVar database [19]. If the disease associated with a variant was not found in the graph, a new node was created for that disease and then connected to the variant.

To further enrich the graph with biomolecular information, we also classified each protein–protein edge into one of two types: transient or permanent, based on whether the physical interaction between the two proteins is transient in time or permanent in time. To classify a protein–protein interaction (PPI) as transient or permanent, we obtained time-course gene expression data from experiments in the GEO database [20] and calculated for each protein pair the time-course co-expression of their two coding genes in each experiment. A PPI was then classified as transient in one experiment if the co-expression of its two corresponding genes in that experiment is less than 0.1; otherwise, the PPI was classified as permanent. An edge connecting two proteins in the graph was then labeled as transient if its corresponding PPI was classified as transient in the majority of experiments; otherwise, it was labeled as permanent. Edges whose corresponding PPIs were neither classified as transient nor permanent, due to lack of co-expression data in GEO experiments, were grouped into a third unlabeled category.

**Figure 1:**
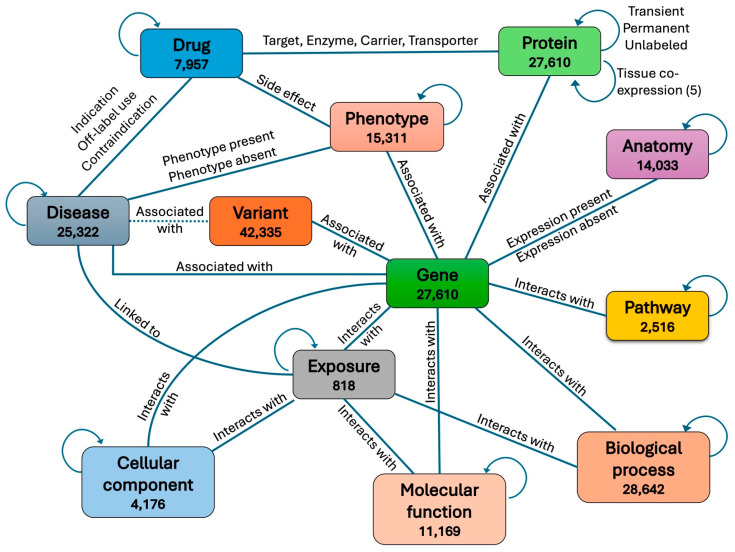
Full graph structure with all 12 node types after including variants. In contrast to the *full* graph, the “basic graph” includes only the 5 node types: gene, protein, variant, pathway and disease. Edges between disease nodes and pathogenic variants, represented here with a dotted link, are masked during training.

We also added a second edge type between every two connected proteins in the graph, this time representing their level of tissue co-expression. We first obtained CAGE (Cap Analysis of Gene Expression) peak data in human from the Fantom5 project [21] and calculated the co-expression level for each pair of genes across different tissues and cell lines. We then labeled the new connection between each protein pair in the graph as “negative co-expression”, “no co-expression”, “low positive co-expression”, “medium positive co-expression”, or “high positive co-expression”, based on the co-expression level of the two corresponding genes. The full graph including all node types and edge types is shown in Figure 1. The count for each edge type in the graph is shown in Table 1.

Initial feature vectors for all node types in the graph were obtained from BioBERT v1.1 [22], a domain-specific language representation model pretrained on large-scale biomedical corpora. For each node in the graph, we used a textual description of the node as input to the BioBERT model to obtain an embedding vector of length 768. For drug and disease nodes, natural language descriptions covering different biomolecular and clinical features, provided by Chandak et al., 2023 [18], were used. The BioBERT output for each drug or disease node was then averaged across all features to obtain the final embedding of a node. For disease nodes that were not part of Chandak’s graph, the name of the disease was used as the input. For variant nodes, the following textual description was used as input to the BioBERT model: “*<variant type> located on chromosome <chromosome number> at position <variant position> with reference allele <reference allele> and alternative allele <alternative allele>*”. For protein nodes, the following textual description was used as input to the BioBERT model: “*Protein encoded by gene <gene symbol>*”. For all other node types, the name of the node provided by Chandak et al. was used.

In addition to BioBERT, two other genomic foundation models were also used to generate an embedding vector for each variant, HyenaDNA [16] and Nucleotide Transformer [17]. HyenaDNA was pretrained on the human reference genome with context lengths up to 1 million tokens at the single nucleotide level. It provides four different models that take as input genomic sequences of maximum lengths 1 Kb, 160 Kb, 450 Kb and 1 Mb. We used the 160 K and 1 M models in our study. Nucleotide Transformer provides several models each trained on a different set of reference genomes. We used the v2-500m-multi-species model (NT-500m) in our study, which is a 500 million-parameter transformer pretrained on 850 genomes from a wide range of species. This model accepts genomic sequences with a maximum length of 12,282 bases (12K).

To obtain an embedding for a missense variant using HyenaDNA or Nucleotide Transformer, we first identified the variant position on the chromosome sequence and obtained the variant’s surrounding sequence using a context window centered at that variant position. The context window length was set to the maximum length accepted by the model, which is 160 K or 1 M for HyenaDNA models and 12,282 (12 K) for Nucleotide Transformer. The extracted sequence was then passed as input to the embedding model after replacing the reference allele at the variant position with the alternative allele. In cases where the variant was located near the edge of the chromosome with the context window extending in that direction outside the chromosome sequence by *n* positions (on one side of the variant), we shifted the context window by a number of *n* positions towards the other side of the variant so that the context window falls completely within chromosome limits.

To obtain an embedding for an insertion variant, we first identified the position where the insertion occurs on the chromosome sequence. We then obtained the variant’s surrounding sequence using a context window centered at that position. Next, the insertion sequence was inserted at the center of the extracted reference sequence. Subsequently, the new sequence, which now included the inserted segment, was equally truncated on each side to preserve its original length while factoring in the insertion. The alternative sequence was then passed as input to the embedding model.

To obtain an embedding for a deletion variant, we first obtained the reference sequence centered at the center of the “segment to be deleted” and of length equal to our set window length plus the length of the “segment to be deleted”. We then removed the deletion segment, which resulted in an alternative sequence of equal length to that of our set window. The alternative sequence was then passed as input to the embedding model. In a similar fashion, we obtained the embedding of an indel variant where a reference sequence was replaced by an alternative sequence by treating it as a deletion followed by an insertion.

We then applied a 2-layer graph convolutional network (GCN) onto the graph, after normalizing the initial embedding for each node using L2-normalization, followed by a 3-layer neural network (NN) operating as a decoder on the node embeddings generated by the GCN. The goal here was to predict disease-specific pathogenicity of variants. To this end, the NN took as input the embedding of a variant node concatenated with the embedding of the disease node for which variant pathogenicity was to be predicted. The initial dimension of an embedding vector for a variant node was either 256, 768 or 1024, depending on the choice of embedding model (HyenaDNA, BioBERT or Nucleotide Transformer, respectively). Every other node type in the graph had an initial embedding dimension of 768 obtained from BioBERT. We set the hidden dimensions of the two GCN layers to d(1) = 256 and d(2) = 64 with a dropout rate of 0.3 applied to each layer. The NN decoder took two node embeddings (variant–disease pair) as input with a total dimension of 2 × 64 = 128. We set the hidden dimensions of the two GCN layers to d(1) = 256 and d(2) = 64 with a dropout rate of 0.3 applied to each layer. The NN decoder took two node embeddings (variant–disease pair) as input with a total dimension of 2 × 64 = 128. We set its hidden layers to d(1) = 64 and d(2) = 32 neurons, with a dropout rate of 0.3 applied to each layer. A sigmoid activation function was then applied to the output neuron to map its output to a value between 0 and 1, representing the final probability for a variant–disease pair to be pathogenic.

For the positive class in our dataset, we paired every pathogenic variant in the graph with each one of its associated diseases. We then concatenated the embeddings generated by the GCN for the two nodes corresponding to each variant–disease pair and passed them as input to the NN. For the negative class, we paired every benign variant in the graph with every disease connected to the gene linked to that variant. We then concatenated the embeddings generated by the GCN for the two nodes corresponding to each variant–disease pair and passed them as input to the NN. Pathogenic variant–disease pair examples were given a label of 1, whereas benign variant–disease pair examples were given a label of 0. To train the model, the full set of positive (pathogenic) and negative (benign) examples were split into 70% for training, 15% for validation, and 15% for testing. We then trained the full GCN+NN model in an end-to-end fashion using a stochastic gradient descent optimizer with a learning rate of 0.001, weight decay of 0.1, and momentum of 0.9. Of note, the edges between disease nodes and pathogenic variant nodes in the graph were masked during training to avoid information leakage. The model state with minimum binary cross entropy loss on the validation set was finally selected for testing.

### 2.2. Neural Network-Based Prediction of Disease-Specific Pathogenicity of Variants

As a baseline for comparing the performance of our graph model on disease-specific prediction of variant pathogenicity, we trained a 3-layer NN alone without the graph. In this approach, instead of using variant and disease embeddings generated by the GCN as input to the NN, we used the initial raw embeddings generated by BioBERT for diseases, and initial raw embeddings generated by either BioBERT, HyenaDNA or Nucleotide Transformer for variants. We then trained the NN with hidden dimensions d(1) = 256 and d(2) = 64, and learning parameters similar to those used with the full GCN+NN model for the task.

### 2.3. Neural Network-Based Prediction of Non-Disease-Specific Pathogenicity of Variants

In the previous graph-based and baseline NN approach, our definition of variant pathogenicity was disease-specific. In other words, we defined pathogenicity as a pairwise attribute of variants and diseases, where a variant–disease pair was classified as pathogenic (positive), if the variant was predicted to cause that disease in particular. On the other hand, a variant–disease pair was classified as benign (negative), if the variant is predicted to not cause that disease in particular.

Here, we again trained a 3-layer NN alone, without the graph, using the same architecture and learning parameters as before. However, this time we used only the variant embedding as input. With this approach, pathogenicity was defined as a variant attribute which was independent of any disease context. This allowed us to test how much signal there was within the raw embedding generated from DNA language models for predicting non-disease-specific pathogenicity of variants and also to further assess the advantage of integrating these embeddings into a relational graph that captures biological dependencies.

### 2.4. Optimizing the Graph Model by Fine-Tuning Nucleotide Transformer on Predicting Variant Pathogenicity

The DNA language models, HyenaDNA and Nucleotide Transformer, that were used to generate variant embeddings for our graph model, have not been pretrained specifically on the task of predicting variant pathogenicity, even in a disease-agnostic manner. Therefore, we hypothesized that fine-tuning the DNA language model on the task of predicting variant pathogenicity would generate better embeddings that capture variant pathogenicity features from raw DNA sequence and that this would consequently improve the performance of our graph model.

To explore this hypothesis, we fine-tuned the Nucleotide Transformer model on the task of predicting non-disease-specific pathogenicity of variants. To do this, we obtained the genomic context sequence for each variant, as in previous sections. Sayeed et al. reported that Nucleotide Transformer’s 100 million-parameter model (NT-100m) outperformed other models when fine-tuned on this task [23]. Thus, we used the same parameters and fine-tuned the NT-100m model using a sequence length of 400 bases.

After down-sampling the benign class to balance it with the pathogenic class, we split the sequence dataset into 70% for training, 15% for validation, and 15% for testing. We then trained the Nucleotide Transformer model for binary classification on small batches of the data over 42 epochs (60,000 steps). We used 4 GPUs with 40 G memory each, a cosine learning rate scheduler with a warmup ratio of 0.1, and learning rate not exceeding 1 × 10^−5^. Lastly, we generated variant embeddings from the fine-tuned model, trained our graph model again on the new embeddings, and compared the performance of our model based on fine-tuned embeddings with its performance without fine-tuning.

### 2.5. Computational Requirement

We used a single NVIDIA A100SXM4 GPU with 40 G memory to train all NN models and graph-based models, except for fine-tuning NT-100m where we used four GPUs of the same model. Models were implemented using Pytorch [24] and Pytorch Geometric [25] deep learning frameworks. 

### 2.6. ClinVar Variants

Our dataset included 42,335 missense variants from ClinVar [19]. We selected only germline variants with their positions provided by ClinVar according to genome assembly GRCh38. Specifically, we focused only on variants labeled as “benign” or “pathogenic”. We discarded all other ClinVar variants, including those with labels “benign/likely benign”, “likely benign”, “pathogenic/likely pathogenic”, “likely pathogenic”, “uncertain significance”, “drug response”, “affects”, “risk factor”, “association”, “protective”, “not provided”, “other”, “no interpretation for the single variant”, “conflicting data from submitters”, and “conflicting interpretations of pathogenicity”. Moreover, in terms of quality of variants, we focused only on those whose review status in ClinVar is either “practice guideline”, “reviewed by expert panel”, “criteria provided, multiple submitters, no conflicts” or “criteria provided, single submitter”. Finally, we selected only variants that are associated with a gene and located on nuclear DNA.

Since most genes have an unbalanced ratio of benign variants to pathogenic variants listed in ClinVar, *during training* we balanced the number of benign variants with the number of pathogenic variants for each gene. More specifically, for every benign variant–disease pair selected from a gene to be added as a negative example to the training set, we selected one pathogenic variant–disease pair from the same gene to be added as a positive example to the training set as well. At the same time, two other variants from the same gene were randomly selected to be included in the validation set and test set, with the two variants selected from different classes (pathogenic vs. benign) when possible.

For our best performing pipeline, we repeated the analysis by splitting ClinVar variants by their submission dates. Specifically, we used all variants submitted up to the end of 2022 for training and validation (split into 80% for training and 20% for validation), and variants submitted during 2023–2024 for testing, thereby simulating a forward-in-time deployment scenario.

### 2.7. Statistical Significance Test and Confidence Intervals

A binomial test with probability of success *p* = 0.5 was used to calculate the *p*-value for each balanced accuracy, after adjusting the number of observed successes by the observed balanced accuracy (i.e., successes = balanced accuracy × number of test cases). 95% confidence intervals for balanced accuracies were calculated using a Wilson score on the observed number of successes adjusted for balanced accuracy as described above. Confidence intervals were also calculated using the bootstrap method of the *scikits* library on the prediction data directly. Both methods produced the same results.

## 3. Results

### 3.1. Predicting Pathogenicity of Missense Variants with a Neural Network Only vs. With Our Graph

Training first the NN on predicting variant pathogenicity in a non-disease-specific manner, allowed us to assess the signal within the raw embedding generated from DNA language models for predicting variant pathogenicity. Table 2 summarizes the results of using the variant embeddings obtained from BioBERT, HyenaDNA and Nucleotide Transformer as input to the NN. Overall, regardless of the source of embeddings for variants, when following this “non-disease-specific approach”, NN could not predict pathogenicity of missense variants. The embeddings generated by DNA language models had no signal to predict variant pathogenicity on their own.

Although NN alone (i.e., without the graph), had some signal when following the “disease-specific approach” (Table 2), the balanced accuracy in predicting pathogenicity of missense variants using our graph (Table 3) was higher than that of NN alone. Our graph model shows up to ~8% improvement in performance compared to the baseline NN model.

Of note, balancing the number of benign variant–disease pairs and pathogenic variant–disease pairs per gene, *during training*, lead to lower performance (Table 3). This is not surprising as this approach was ensuring that the model could not exploit the skewed distribution of pathogenic and benign variants within each gene.

### 3.2. Predicting Disease-Specific Variant Pathogenicity with a Basic Graph, Rather than Full Graph

Our full graph includes 12 node types of diverse biological and clinical nature. Some of these node types may be more important for predicting variant pathogenicity than others. For example, we expected nodes carrying information at the molecular level such as genes and pathways to be more essential for predicting variant pathogenicity than other nodes carrying information at the higher clinical and environmental level. To explore this, we tested how our graph model performed if only five basic nodes were included in the graph (variants, genes, proteins, pathways, and diseases). As shown in Table 4, our model operating on the basic graph performs on par with the model operating on the full graph, suggesting that these five basic elements of the graph are more essential for variant pathogenicity prediction than other higher-level elements.

Of note, training our model on the basic graph using a much shorter genomic sequence length of 400 bases for obtaining variant embeddings, did not affect model performance.

### 3.3. Improving Graph Model Performance with Fine-Tuned Nucleotide Transformer Embeddings

To further optimize the performance of our basic graph model on missense variants, we then used variant embeddings obtained from the Nucleotide Transformer model with and without fine-tuning (on predicting pathogenicity for missense variants). Our disease-specific graph model showed a 10.7% increase in prediction accuracy when using variant fine-tuned embeddings (Table 5). Variants assigned to the training, validation and test partitions during fine-tuning stage were placed as part of the corresponding sets during the graph learning stage as well. This was carried out to maintain consistency across stages and ensure fail evaluation without label leakage. Fine-tuning was performed using only variant sequence information, without incorporating additional data such as disease annotations, to ensure that the model’s performance reflects its ability to generalize from sequence features alone.

## 4. Discussion

Previous studies for predicting variant pathogenicity have generally followed non-disease-specific approaches. They have either focused on predicting variant pathogenicity targeting variants in both protein-coding regions and non-coding regions [23], or on predicting on all types of variants in coding regions [5]. To our knowledge, our study is the first to focus on predicting pathogenicity for missense variants, which appear to represent the most problematic type of variants for different reasons.

First, predicting pathogenicity of variants in coding regions is more challenging than predicting pathogenicity across coding and non-coding regions. Pathogenic variants are more likely to occur in coding regions than benign variants, and benign variants are more likely to occur in non-coding regions than pathogenic variants. Hence, the task of predicting pathogenicity is less complex when focusing on both coding and non-coding regions, rather than when restricting the analysis to coding DNA. In addition, focusing on missense variants represents a major clinical challenge, as a significant proportion of missense variants identified in clinical settings are classified as VUS. Notably, most VUS are missense variants. This is a substantial problem: approximately 41% of variants detected by clinical diagnostic tests in protein-coding regions are classified as VUS, and only about 7% of these are later reclassified based on clinical and experimental evidence [14].

Our graph-based approach integrates variant genomic context into a relational structure that captures biological dependencies from multiple sources. This goes beyond the conventional task of simply predicting variant pathogenicity in a disease-agnostic manner. By tailoring variant effect predictions to specific disease contexts, our approach integrates disease-specific domain knowledge, making our predictions more clinically relevant. According to our results, a neural network alone was unable to predict non-disease-specific pathogenicity of missense variants, regardless of the source for obtaining variant embeddings (BioBERT, HyenaDNA or Nucleotide Transformer).

When introducing disease-specificity, a neural network alone was able to predict pathogenicity of missense variants, albeit with a low accuracy (62%, Table 2). In contrast, the accuracies of predictions exceeded 70% when a neural network was combined with our graph (Table 3). This was true, irrespective of the sources for variant embeddings (BioBERT, HyenaDNA or Nucleotide Transformer). Moreover, pruning the graph down to the five basic node types (variants, genes, diseases, proteins, and pathways), while excluding all other node types, had no significant impact on performance (Table 4). This suggests that the corresponding five elements (gene, protein, variant, pathway, and disease) may be treated as the backbone for future prediction models and be further exploited for performance optimization. Similarly, fine-tuning the basic graph improved the disease-specific graph model performance to ~86% (Table 5). For example, our model correctly classified all 14 pathogenic variants for Charcot-Marie-Tooth disease, despite their being spread in different genes (LMNA, MFN2 and GARS1) [26].

It is worth noting that when we split the ClinVar dataset based on submission date, the performance of the model was very similar, both at the fine-tuning stage as well as the final graph learning stage. The final balanced accuracy was 86.5% compared with the 85.6% in random splitting used throughout the paper. Similarly, when we collapsed the PPI linkage to one type of edge, the balanced accuracy remained unchanged to 85.74%. The consistent performance across both cases demonstrate that our pipeline is resilient to sampling variation and graph construction bias. Performance metrics for all our analyses, including 95% Confidence Intervals, are provided in Table 2, Table 3, Table 4 and Table 5. The *p*-values are not included in the tables, as they are all nearly zero (<1 × 10^−5^). The confusion matrices are provided in the Supplemental Section, along with additional details for our model (Supplemental Tables S1–S3).

Given the high sensitivity (Recall: 91%) and negative predictive value (NPV: 90%) of the disease-specific basic graph model with fine-tuning, this can have clinical implications. Our model can be used for bioinformatic tool as part of the American College of Medical Genetics (ACMG) Guidelines for variant interpretation. As mentioned above, most genetic variants are currently classified as VUS based on ACMG criteria. The standard interpretation process factors in outputs of different bioinformatic tools. Popular bioinformatic tools, such as CADD [27] or PolyPhen-2 [28], rely on a different methodology than our model. Our approach can thus offer complementary bioinformatic evidence in the process of variant interpretation. As whole genome sequencing (WGS) becomes a first-tier test in clinical practice, and potentially a complement test for newborn screening (NBS) programs, our approach can contribute towards the classification of WGS variants in a disease-specific manner.

Introducing disease specificity in variant interpretation appears to be a promising approach to explore further. This is particularly true within our knowledge graph, which was shown to largely improve accuracy of the predictions. However, our approach is limited to variant interpretation for known diseases and cannot be used for variants in novel genes which are not yet linked to diseases. An alternative strategy of training would be to exclude entire genes from training for validation or testing, or to block gene–disease links at training time. However, such approaches would not reflect real-world scenarios. In practice, the set of genes is fixed, and the model must learn meaningful representation (embeddings) for them, including the range of their diseases and the types of variants that cause them. (The latter is not generalizable across genes, as highlighted by the higher performance of our disease-specific approach). When a new variant arises in a clinical setting, it would be mapped to one of the known genes within the graph, and the model would predict its association with one of the known disease nodes. Our focus in a clinical setting is to interpret variants within genes known to be associated with one or more genetic diseases. To this end, our design allows for the model to learn which diseases are associated with a specific gene through the existing gene–disease link. At the same time, by including an equal number of positive and negative samples (variants) per gene relative to its known diseases, we ensure that the algorithm is not exploiting unequal distribution of variant–disease association within a gene.

In terms of technical learning lessons derived from our study, we should highlight the importance of window size, when generating variant embeddings from genomic sequence. This refers to the window size applied on both sides of the variant to read its context sequence. Theoretically, a large window size can potentially capture long-range genomic context of importance to a variant. However, it may also introduce unwanted noise affecting the process of discriminating benign variants from pathogenic variants, especially when these variants are in close proximity from each other. An extremely large window size may even render the model unable to learn. To address this question, we used two different DNA language models to generate variant embeddings from genomic sequence, namely, HyenaDNA and Nucleotide Transformer, with different context window sizes: 160 K and 1 M for HyenaDNA, and ~12 K for Nucleotide Transformer. Although our results, overall, showed that varying the window size did not significantly affect performance, reducing the context size was particularly important for the fine-tuning task.

In addition, our findings suggest that simply embedding the variant type, location, reference allele and alternative allele using BioBERT can achieve similar performance to that of the DNA language models. DNA language models have recently gained popularity, as they can be trained on a large number of genomes from different species. Feng et al. showed that DNA language models may exhibit superior performance for certain genomic tasks [29]. However, Sayeed et al. evaluated the performance of these models in predicting the pathogenicity of single nucleotide variants (SNVs) and found that these models may not perform as well as the other non-DNA language models on this specific task [23]. Our findings support these conclusions and do not provide evidence for a role of DNA language models for variant interpretation.

Overall, in our two-staged training scheme, a DNA language model was fine-tuned at the nucleotide level, and then, the resulting embeddings were integrated into a heterogenous graph to predict disease-specific variant effect using GNNs. While our approach uses DNA sequence-derived representations and a biomedical knowledge graph, multi-stage training has broad applications. For example, Tan et al. [30] pretrained a GNN on synthetic epidemic contact networks, using approximate likelihoods, and subsequently fine-tuned it with more precise labels to infer superspreaders. The approach by Tan et al. differs from ours in terms of the type of graph, task, and supervision. However, the two approaches share the principle of progressively refining network representations through staged learning. Multi-phase training strategies capture latent relational dependencies in complex networks and can be useful in different clinical contexts.

## 5. Conclusions

In this study, we implemented a novel graph-based approach for predicting disease-specific pathogenicity of missense variants. We utilized a comprehensive biomedical knowledge graph integrating genetic variants with 11 types of entities covering different levels of human biology. We also made use of multiple embedding models to integrate biological features for every type of node in the graph, including genomic sequence information for all variants. Our graph model, optimized for predicting disease-specific pathogenicity of missense variants, shows high performance with an accuracy reaching ~86% when variant embeddings are fine-tuned specifically for this task. Our study opens the door to new approaches in studying variant pathogenicity in a way that is more relevant to clinical practice by introducing disease specificity in a graph-based paradigm.

## 6. Future Directions

Our balancing the benign class and pathogenic class per gene, although crucial to prevent class imbalance bias, lead to lower number of variants available per gene, and negatively affected our model performance (Table 3). By increasing the sample size of variants available for a given gene, our model would be able to better capture gene-specific domain knowledge and potentially increase the accuracy of its predictions for certain genes/diseases. Training our graph model using variants from large locus-specific/gene-specific databases could lead to even higher accuracies for the corresponding variant pathogenicity predictions. Over the next months, we are planning to make our model available in a user-friendly manner at MD-YOU.COM. Meanwhile, we would be happy to be contacted by moderators of different locus-specific databases and collaborate towards optimizing our model’s performance for specific genetic conditions where large datasets of clearly labeled variants (benign vs. pathogenic) are available.

## Figures and Tables

**Table 1:** Count for each edge type present in the full graph.

Edge Type	Count
Drug–Drug (synergistic interaction)	2,672,628
Anatomy–Gene (expression present)	1,518,203
Protein–Protein (transient PPI)	376,344
Variant–Gene (associated with)	326,096
Protein–Protein (permanent PPI)	206,844
Disease–Variant (associated with) [*masked during training*]	202,093
Protein–Protein (low positive co-expression PPI)	185,140
Disease–Phenotype (phenotype present)	176,383
Protein–Protein (no co-expression PPI)	163,004
Bioprocess–Gene (interacts with)	144,805
Protein–Protein (medium positive co-expression PPI)	125,136
Bioprocess–Bioprocess (parent–child)	105,772
Disease–Gene (associated with)	97,338
Protein–Protein (negative co-expression PPI)	88,476
Cellular component–Gene (interacts with)	83,402
Disease–Disease (parent–child)	75,618
Molecular function–Gene (interacts with)	69,530
Drug–Effect/Phenotype (side effect)	64,784
Protein–Protein (unlabeled PPI)	58,962
Pathway–Gene (interacts with)	42,646
Protein–Protein (high positive co-expression PPI)	42,228
Phenotype–Phenotype (parent–child)	37,472
Contraindication (contraindication)	33,984
Anatomy–Anatomy (parent–child)	28,064
Protein–Gene (associated with)	27,610
Molecular function–Molecular function (parent–child)	27,148
Anatomy–Gene (expression absent)	19,887
Drug–Protein (target)	16,380
Indication (indication)	10,061
Cellular component–Cellular component (parent–child)	9,690
Drug–Protein (enzyme)	5,317
Pathway–Pathway (parent–child)	5,070
Exposure–Exposure (parent–child)	4,140
Phenotype–Gene (associated with)	3,330
Drug–Protein (transporter)	3,092
Off-label use (off-label use)	2,759
Exposure–Disease (linked to)	2,311
Exposure–Bioprocess (interacts with)	1,625
Disease–Phenotype (phenotype absent)	1,345
Exposure–Gene (interacts with)	1,212
Drug–Protein (carrier)	864
Exposure–Molecular function (interacts with)	45
Exposure–Cellular component (interacts with)	10

**Table 2:** Balanced accuracy in predicting pathogenicity of missense variants using a neural network without the graph.

Variant Type	Variant Embedding Source			
	BioBERT	HyenaDNA 160 K	HyenaDNA 1 M	NT-500m 12 K
Non-disease-specific	50.0 (95% CI: 47.9, 52.1)	50.2 (95% CI: 48.1, 52.4)	49.9 (95% CI: 47.7, 52.0)	50.1 (95% CI: 48.0, 52.3)
Disease-specific	62.3 (95% CI: 61.3, 63.3)	62.2 (95% CI: 61.2, 63.3)	50.0 (95% CI: 48.9, 51.1)	62.0 (95% CI: 61.0, 63.0)

**Table 3:** Accuracy in predicting disease-specific pathogenicity using the graph model.

Variant Type	Variant Embedding Source			
	BioBERT	HyenaDNA 160 K	HyenaDNA 1 M	NT-500m 12 K
Missense variants	76.7 (95% CI: 75.8, 77.6)	79.8 (95% CI: 78.9, 80.7)	74.4 (95% CI: 73.5, 75.4)	75.2 (95% CI: 74.3, 76.2)
Missense variants (balanced per gene)	70.8 (95% CI: 69.8, 71.7)	70.9 (95% CI: 69.9, 71.9)	70.6 (95% CI: 69.6, 71.5)	70.2 (95% CI: 69.3, 71.2)

**Table 4:** Accuracy in predicting disease-specific pathogenicity using a basic graph.

Variant Type	Variant Embedding Source			
	BioBERT	HyenaDNA 160 K	HyenaDNA 1 M	NT-500m 12 K
Missense variants	76.5 (95% CI: 75.5, 77.4)	77.1 (95% CI: 76.2, 78.0)	77.9 (95% CI: 77.0, 78.8)	78.4 (95% CI: 77.6, 79.3)
Missense variants (balanced per gene)	68.4 (95% CI: 67.3, 69.3)	69.7 (95% CI: 68.7, 70.7)	70.3 (95% CI: 69.3, 71.2)	67.5 (95% CI: 66.5, 68.5)

**Table 5:** Accuracy in predicting pathogenicity of missense variants using the basic graph with variant embeddings obtained from NT-100m with and without fine-tuning.

Variant Type	Disease-Specific Graph Model	
	NT-100m	Fine-tuned NT-100m
Missense variants	74.9 (95% CI: 74.0, 75.8)	85.6 (95% CI: 84.8, 86.3)

## Data Availability

The genetic variant data used in this study are available in ClinVar. Over the next months, we plan to make the model available in user friendly manner through a web solution that will be available at MD-YOU.COM. In the meantime, please contact us by email for interpretation of your variants.

## Supplementary Materials

The following supporting information can be downloaded at https://www.mdpi.com/article/10.3390/bioengineering12101098/s1, Table S1. Confusion matrix for the basic graph with variant embeddings from the NT-100m model without fine-tuning. Table S2. Confusion matrix for the basic graph with variant embeddings from fine-tuned NT-100m model. Table S3. Data split and training epochs for fine-tuning the NT-100m model and training the basic graph with variant embeddings from NT-100m.
