# Supplementary material for: Disease-Specific Prediction of Missense Variant Pathogenicity with DNA Language Models and Graph Neural Networks
Source: Bioengineering (Basel). 2025 Oct 13;12(10):1098. doi: 10.3390/bioengineering12101098 (PMC12562010; doi:10.3390/bioengineering12101098)
Supplement: Supplementary file 1 [file bioengineering-12-01098-s001.zip › bioengineering-3862539-supplementary.pdf]

Supplemental Section

Supplemental Table S1. Confusion matrix for the basic graph with variant embeddings from the NT-100m model without fine-tuning.

	Predicted positive	Predicted negative
Positive	3646	437
Negative	1694	2598

Supplemental Table S2. Confusion matrix for the basic graph with variant embeddings from fine-tuned NT-100m model.

	Predicted positive	Predicted negative
Positive	3772	395
Negative	831	3465

Supplemental Table S3. Data split and training epochs for fine-tuning the NT-100m model and training the basic graph with variant embeddings from NT-100m.

	Fine-tuned NT-100m			Disease-specific graph model					
				NT-100m			Fine-tuned NT-100m		
	Train	Valid	Test	Train	Valid	Test	Train	Valid	Test
Percentage	70	15	15	70	15	15	70	15	15
Count	19,928	4270	4272	38,936	8336	8375	38,480	7993	8463
Total	28,470			55,647			54,936		
